# Room-Temperature Plasmon-Assisted Resonant THz Detection in Single-Layer Graphene Transistors

**DOI:** 10.1021/acs.nanolett.3c04300

**Published:** 2024-01-02

**Authors:** José M. Caridad, Óscar Castelló, Sofía M. López Baptista, Takashi Taniguchi, Kenji Watanabe, Hartmut G. Roskos, Juan A. Delgado-Notario

**Affiliations:** † Department of Applied Physics, University of Salamanca, Salamanca 37008, Spain; ‡ Unidad de Excelencia en Luz y Materia Estructurada (LUMES), Universidad de Salamanca, Salamanca 37008, Spain; § Research Center for Materials Nanoarchitectonics, National Institute for Materials Science, 1-1 Namiki, Tsukuba 305-0044, Japan; ∥ Research Center for Electronic and Optical Materials, National Institute for Materials Science, 1-1 Namiki, Tsukuba 305-0044, Japan; ⊥ Physikalisches Institut, Johann Wolfgang Goethe-Universität, Max-von-Laue-Str. 1, Frankfurt am Main D-60438, Germany

**Keywords:** terahertz, resonant detection, plasmons, graphene, two dimensional materials

## Abstract

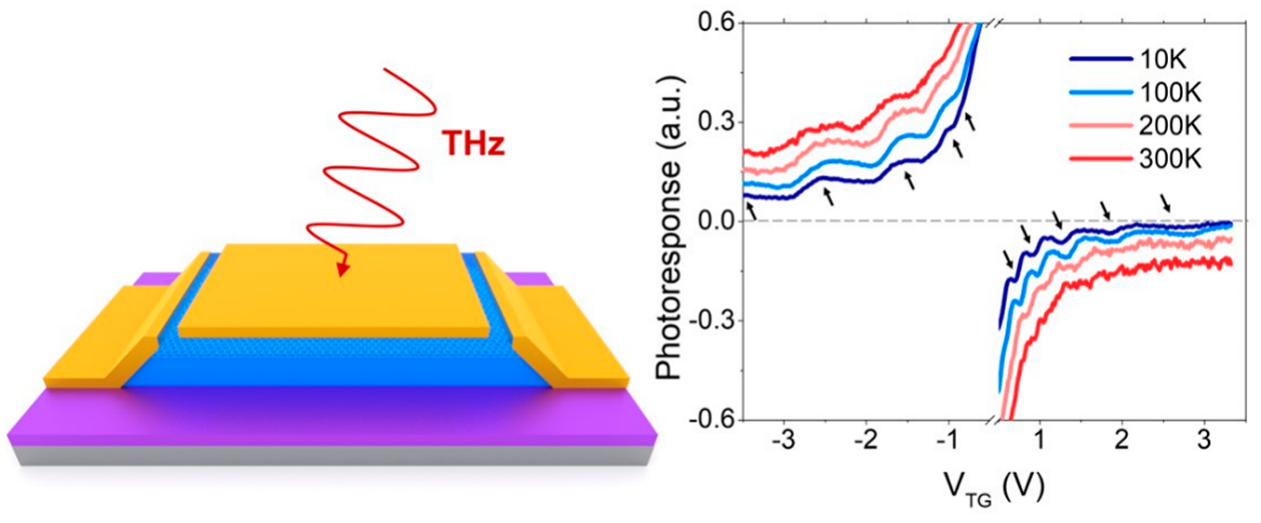

Frequency-selective or even frequency-tunable terahertz (THz) photodevices are critical components for many technological applications that require nanoscale manipulation, control, and confinement of light. Within this context, gate-tunable phototransistors based on plasmonic resonances are often regarded as the most promising devices for the frequency-selective detection of THz radiation. The exploitation of constructive interference of plasma waves in such detectors promises not only frequency selectivity but also a pronounced sensitivity enhancement at target frequencies. However, clear signatures of plasmon-assisted resonances in THz detectors have been revealed only at cryogenic temperatures so far and remain unobserved at application-relevant room-temperature conditions. In this work, we demonstrate the sought-after room-temperature resonant detection of THz radiation in short-channel gated photodetectors made from high-quality single-layer graphene. The survival of this intriguing resonant regime at room temperature ultimately relies on the weak intrinsic electron–phonon scattering in monolayer graphene, which avoids the damping of the plasma oscillations present in the device channel.

Terahertz (THz) radiation (0.1–10 THz) has a strong perspective in a wide range of different applications, including metrology and characterization of nanomaterials,^1^ upcoming 6G wireless communications,^2^ noninvasive imaging,^3^ biosensing,^4^ high-resolution spectroscopy,^5^ together with many others.^6,7^ An emerging and important research area within THz technology is the study of novel, efficient, and functional photodetectors operating at these frequencies.^8^ The majority of photodetectors reported to date (if not all), including sensors made of many different nanomaterials,^9−15^ operate either in broadband mode (i.e., without being selective to a given frequency) at room temperature or over narrow fixed frequency bands (i.e., without being frequency tunable), for example, by embedding antennas in the detector. Frequency-tunable THz photodetectors working at atmospheric conditions are therefore unavailable so far, despite being desirable components to (i) boost the performance of some applications at specific and selected THz wavelengths^16^ and (ii) provide new functionalities such as selective sensing, frequency mixing, multiplication, and modulation as well as nanoscale confinement of light.^17^

One of the most prominent ideas to design tunable and selective THz photodetectors, originally introduced by M. Dyakonov and M. Shur more than two decades ago, predicts that two-dimensional (2D) gated FETs may exhibit a resonant response to electromagnetic THz radiation at discrete plasma oscillation frequencies of the 2D electrons in the device channel.^17^ In this pioneering proposal, the resonant operation of field-effect transistor (FET) photodetectors is univocally defined by a quality factor, *Q* = ωτ, which must be much larger than unity (*Q* = ωτ ≫ 1, where ω = 2*πf* with *f* being the frequency of the incoming radiation and τ the momentum relaxing scattering time of charge carriers in the system, respectively). In other words, resonant THz photodetection should arise in plasmonic FETs at any temperature, when a negligible damping of the plasma waves occurs in the channel. In such conditions, the device channel acts as a tunable plasmonic cavity with a set of multiple resonances defined by the incoming frequency, the device length, and the density of charge carriers in the system.^17^ This exotic regime is in clear contrast to the more commonly observed and studied broadband (nonresonant or overdamped) case,^18−23^ characterized by *Q* ≪ 1, with plasmons being strongly damped in the channel and even decaying long before reaching the other side of the plasmonic cavity.

To date, several experimental studies have attempted to demonstrate resonant THz detection in different 2D electron gases systems with varying levels of success. Convincing signatures of plasmon resonances, including the appearance of frequency-dependent oscillations in the zero-bias photoresponse of the system w.r.t. the carrier density, have been identified at cryogenic temperatures in FET devices made of some high-quality semiconductors such as III–V materials^24−27^ and bilayer graphene.^28^ However, such features vanish rapidly when operating above cryogenic temperatures and long before reaching room temperature. This fact notably limits the potential use of resonant THz photodetectors for real-life applications.^6,7^

In this Letter, we demonstrate room-temperature THz detection in FET devices made of high-quality, single-layer graphene. In particular, we show how the characteristic frequency-dependent oscillations in the photoresponse of monolayer graphene FETs are largely tunable with the density of charge carriers in the device (i.e., with the applied top gate voltage), and these unique fingerprints of the resonant detection are furthermore visible from cryogenic up to room temperature. The fact that these robust signatures persist up to 300 K in our devices can be directly ascribed to the weak acoustic phonon scattering in monolayer graphene, which leads to large carrier mobility values in the material even at elevated temperatures.^29,30^ In other words, as shown below, the resonant condition *Q* ≫ 1 is also fulfilled in high-quality single-layer graphene FET detectors at room temperature.

In order to observe plasmonic resonant THz detection, we fabricated a short-channel (length *L*_ch_ = 6 μm) dual-gate, high-mobility, single-layer graphene FET device (Figure 1a) by using a state-of-the-art dry-stacking technique^22^ to encapsulate a mechanically exfoliated single-layer graphene sheet in between two thin hexagonal boron nitride (hBN) flakes. The graphene was then side-contacted to Cr/Au (3.5/50 nm) metallic electrodes acting as drain and source contacts. In addition, a metal top-gate electrode covering most of the FET channel (*L*_TG_ = 4.8 μm) was defined on the device, together with a coupled bow tie antenna between top-gate and source electrodes. This antenna ensures an efficient rectification of the incoming THz radiation for a large range of frequencies via gate-to-source coupling (additional fabrication details are shown in Supporting Information, Note 1).

**Figure 1:**
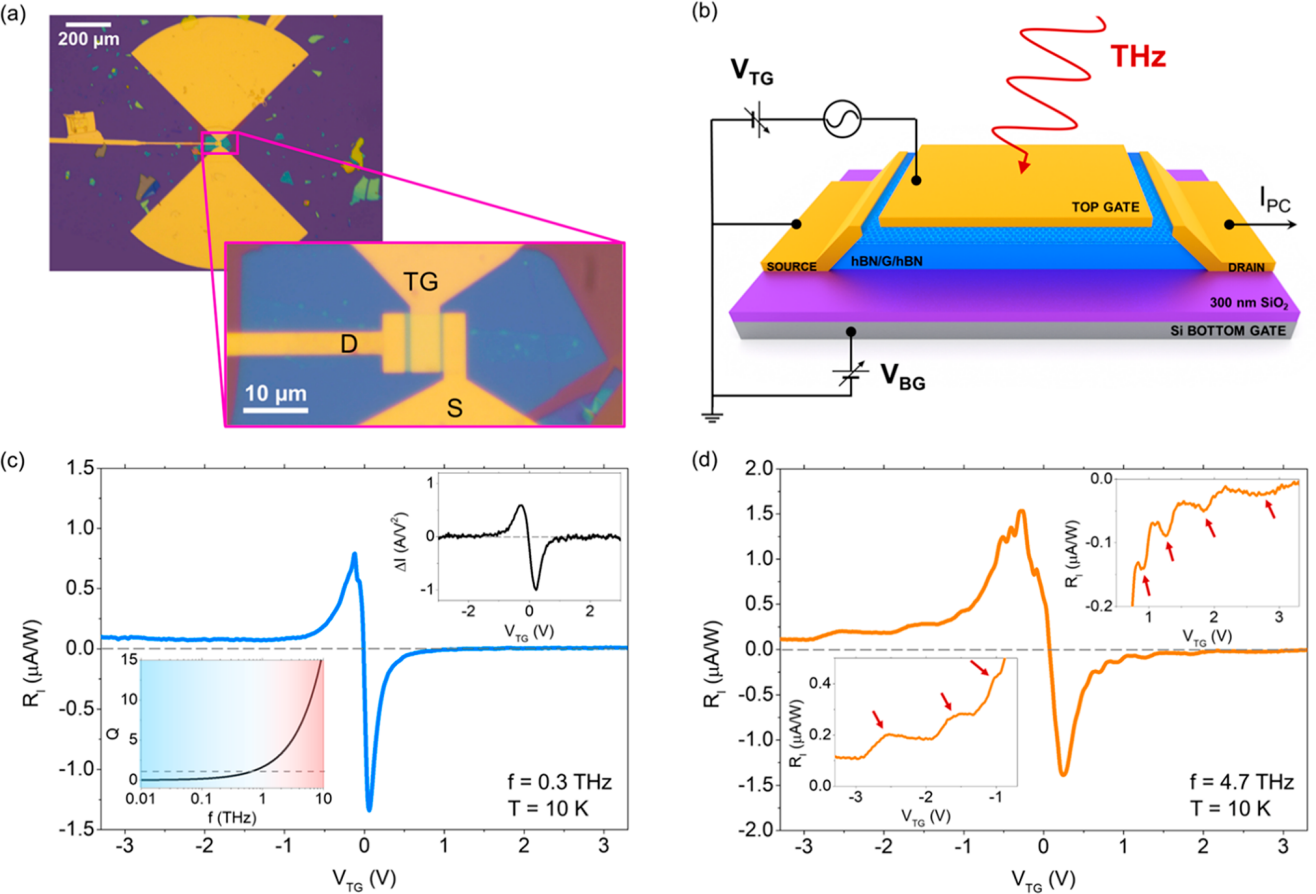
Graphene-based resonant THz photodetector. (a) Optical images of the graphene THz detector with a bow-tie antenna coupled between source and top gate electrodes. The bottom image shows a zoomed view of the device with source (S), top gate (TG), and drain (D) electrodes labeled. (b) Schematic 3D view of the zero-bias photocurrent measurements of the device. (c) Current responsivity, *R*_I_, as a function of the top gate voltage, *V*_TG_, measured at 0.3 THz. The upper-right inset shows the current responsivity expected from the DC conductivity, following the phenomenological formula^21^ Δ*I* = −d*σ*/d*V*_TG_. The bottom-left inset shows the evolution of the *Q* factor in our device as a function of the excitation frequency. The dashed line corresponds to *Q* = 1. Highlighted areas in blue and red indicate the frequencies ranges in which our device operates the overdamped or weakly damped regimes, respectively. (d) *R*_I_ as a function of the top gate voltage measured at 4.7 THz. Inset panels show zoomed in areas of the recorded current responsivity for electron (upper-right) and hole (bottom-left) carriers. Responsivity resonances are highlighted by red arrows in these insets. Here, we note that if the current responsivity is calculated as *R*_I_ = *I*_PC_*S*_T_/*PS*_D_, where *S*_T_ is the THz beam spot area and *S*_D_ is the detector active area, the device performance would reach larger maximum values of ∼0.29 A/W at 0.3 THz and ∼1.8 mA/W at 4.7 THz.

Transport and zero-bias photocurrent measurements in our device were performed in a closed-cycle cryostat, with the chamber temperature varying from 10 K up to 300 K. We employed two different THz sources to perform the photocurrent experiments. First, a sub-THz source was used to undertake measurements at a frequency of 0.3 THz, and then, a quantum cascade laser was used to undertake measurements at frequencies in a range between 2.5 THz up to 4.7 THz (more information about the photocurrent setup can be found in refs (21 and 22)).

First, we measured the transport characteristics of our graphene FET via electrical measurements from 10 to 300 K (see Supporting Information Note 2). We extracted average mobilities, μ, in the device exceeding 70000 cm^2^ V^–1^ s^–1^ for both electron and hole carriers at low temperatures (10 K). Such values remain high, above 60000 cm^2^ V^–1^ s^–1^, even at room temperature (Supporting Information Note 2 contains the measured electrical data as well as details to calculate the carrier mobility). We further estimated the momentum-relaxing scattering time of charge carriers in the device τ to lie between 0.29 and 0.23 ps at 10 K and room temperature, respectively. This is calculated with the relation τ = *mμ*/*e*, where *e* is the elementary charge and *m* is the effective mass of carriers in single-layer graphene. The latter is given for single-layer graphene by *m* = *ℏk*_F_/*v*_F_, where *v*_F_ is the Fermi velocity, *ℏ* is the reduced Plank constant, and *k*_F_ the Fermi wave vector (, with *n* as the carrier density).

We carried out zero-bias (i.e., zero source-drain potential, *V*_DS_) photocurrent measurements at different THz frequencies (Figure 1b). First, we studied the photoresponse of the detector at 10 K for an incoming THz frequency of 0.3 THz. The current responsivity of the device, *R*_I_ = *I*_PC_/*P*, is shown in Figure 1c, with *P* being the incoming power of the THz radiation and *I*_PC_ the measured photocurrent at the drain contact. It is worth noting that, at this radiation frequency, the quality factor value *Q* is below ∼0.5, and thus the photodetector operates in the overdamped regime (see bottom inset of Figure 1c, blue shadowed region). The experimental photoresponse exhibits an antisymmetric shape with respect to the applied top gate potential, which flips its sign at the charge neutrality point (CNP). Such trends, together with the appearance of the maxima and minima values of the photocurrent near the CNP and a vanishing photocurrent at large gate voltages, result from the ambipolar charge transport in graphene and agree with previous published works reporting nonresonant photodetection in the literature.^18−22^ We further highlight that the line shape of the measured current response w.r.t. the gate potential follows closely the trend predicted by theory,^21^ Δ*I* = −d*σ*/d*V*_TG_ (see upper inset Figure 1c), with Δ*I* being the expected photocurrent and σ the DC channel conductivity. The qualitative agreement between both experimental and theoretical curves, with the only sign reversal occurring at the CNP, indicates that the rectified photocurrent in the device is predominantly generated via the so-called plasmonic Dyakonov–Shur (DS) mechanism.^17,20^ Minimal discrepancies from the DS theory appear at large negative top gate voltages, where the experimental current responsivity shows a rather small responsivity offset ∼0.1 μA/W (an order of magnitude lower than the maximum *R*_*I*_ measured), instead of the zero-photocurrent value expected from a pure plasmonic DS mechanism at large gate bias conditions. Such behavior may result from an additional rectification effect occurring due to the presence of pn junctions at the metal–graphene contact.^20,21^

Next, we measured the photoresponse of the device at 10 K but at a higher frequency, 4.7 THz (Figure 1d). The quality factor at this radiation frequency is characterized by *Q* ≫ 1 (*Q* ≈ 8.6), and thus the device operates in the resonant (weakly damped) regime (see Figure 1c, bottom inset). Intriguingly, the current photoresponse recorded at this higher frequency exhibits not only the characteristic antisymmetric line shape with respect to the applied gate voltage (similar to the broadband case depicted in Figure 1c), but also marked oscillations on both electron and hole sides emerged (see arrows in the top-right and bottom-left insets in Figure 1d, respectively). Such oscillations, which are dependent on the carrier density, constitute the hallmark of resonant operation in a FET photodetector.^17,24−28^ They are the result of plasmon resonances occurring in the graphene channel because of the reflection of the plasma waves at the end of the channel and the interference of both reflected and incoming waves. Under such conditions, the graphene device acts like a Fabry–Perot resonant cavity for propagating graphene plasmons under external THz excitation. The multiple ridges presented in *R*_I_ are the result of the crossover from destructive to constructive interferences of the incoming and reflected waves. Subsequently, peaks represent waves with a number of oscillation modes that are by one higher or lower than the neighboring crests. The mode number is tunable with both the length of the top gate (*L*_TG_) and the density of the charge carriers (controlled via the applied gate voltage, *V*_TG_) in the system.^28^ Importantly, the intensity of such resonances strongly depends on the plasmonic cavity length (*L*_TG_) and the plasmon propagation length (*L*_P_, which is larger than the *1/e*-decay length *L*_d_ = *sτ* of the plasma wave and depends on the signal-to-noise ratio at which small modulations of *R*_*I*_ can still be detected), leading to two different scenarios.^17^ When *L*_TG ≲_*L*_P_, propagating plasmons can reach the end of the channel before a total decay, creating interferences between the incoming and reflected waves at least at the end of the channel, if not along its total length (see Supporting Movie 1). Such a case gives rise to different characteristic resonant modes as a function of the carrier density or the incoming frequency. Conversely, if *L*_TG_ ≫ *L*_P_, propagating plasmons in the system decay before reaching the end of the cavity (see Supporting Movie 2), giving rise to a rectified photocurrent indistinguishable to the one expected in the nonresonant scenario.^17^

For completeness, we additionally measured the photoresponse at different frequencies (range 2.5 THz–4.7 THz), all within the resonant regime or weakly damped scenario (*Q* ≫ 1). Figure 2a highlights the evolution of the photoresponse oscillations within this frequency range. For simplicity and clarity, we present the normalized current responsivity, *R*_*I*_^*N*^, with respect to the photocurrent maximum observed close to the CNP. Interestingly, the current photoresponse as a function of the gate voltage exhibits oscillations at all of these measured frequencies, but the visible number of oscillations strongly depends on the THz frequency. In particular, the number of peaks decreases when lowering the excitation frequency. Further frequency-dependent measurements can be found in Supporting Information Note 3.

**Figure 2:**
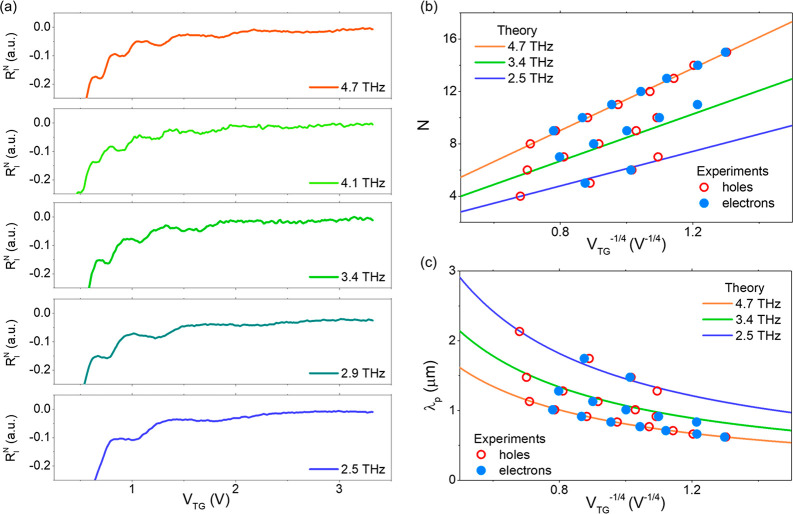
Frequency dependence of resonant THz photodetection. (a) Normalized current responsivity, *R*_*I*_^*N*^, as a function of the top gate voltage at the electron side for different frequencies in the range 2.5 THz–4.7 THz (*Q* ≫ 1 in the device for all these frequencies). All measurements in these five panels were performed at 10 K. In this panel, the measured current responsivity is normalized (*R*_*I*_^*N*^) with respect to the photocurrent maximum recorded close to the CNP for an easier comparison of all recorded data at the different frequencies. (b) Resonant mode number, *N*, of the local minima in the *R*_*I*_^*N*^ curves and (c) corresponding plasmon wavelength, λ_p_, as a function of *V*_TG_^–1/4^ for three selected frequencies from panel (a). Solid lines correspond to the calculated theoretical dependence following eq 4, and symbols represents the extracted values from experiments.

The observed oscillations of the current photoresponse when sweeping *V*_TG_ were further analyzed in the following way. Excited plasmons in gated two-dimensional systems follow the linear dispersion law, ω = *sk*, where *s* is the plasma wave velocity, and *k* is the real part of the angular wavenumber.^24,25^ The plasma wave velocity is defined as
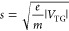
1

And resonances should emerge when the real part of the wavenumber is given by
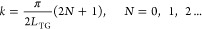
2

Importantly, the effective mass, *m*, in single-layer graphene^18,31^ is dependent on the applied gate voltage as  (in the former expression, *C*_ox_ is the thin-oxide gate capacitance per unit area, and *v*_F_ is the Fermi velocity of the charge carriers). Thus, by replacing *m* into eq 1, the plasma wave velocity in monolayer graphene can be rewritten as
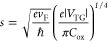
3

Then, using the plasmon dispersion law with eqs 2 and 3, one can easily deduce the relation between the resonant mode number, *N*, and the applied gate voltage, *V*_TG_, for single-layer graphene:
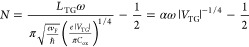
4

Following eq 4, *N* is expected to have a linear dependence with ω and *V*_TG_^–1/4^. We verified that the experimental resonant peaks appearing at all measured frequencies in our device (Figure 2a) follow the predicted *V*_TG_^–1/4^ dependence. In particular, Figure 2b shows the extraordinary agreement between the calculated theoretical dependence *N*(ω,*V*_TG_) given by eq 4 and the values of *N* extracted from the experimental data. We note that, in comparison with systems with parabolic bands,^24−26^ graphene’s linear energy-momentum results in a notably distinct dependence of *N* with the applied voltage (systems with parabolic energy bands exhibit a relation dependence of *N* ∝ *V*_TG_^–1/2^ instead^28^). In consequence, the first resonant modes in our single-layer graphene THz detector (*N* < 6) are not accessible in the recorded *V*_TG_ range at the highest measured frequency 4.7 THz due to the *V*_TG_^–1/4^ dependence of *N* introduced in eq 4. For instance, at 4.7 THz, the resonant mode *N* = 2 is expected to occur for gate potentials larger than 250 V, values that are not reachable in common experimental devices. Resonant modes below 6 are experimentally accessible in our device only when decreasing the excitation frequency down to 2.5 THz (see Figure 2b).

The observed resonant modes in the THz photoresponse can be further utilized to extract significant information on the propagating graphene plasmons.^23,28,32^ Such information includes the plasmon lifetime (τ_p_) and plasmon wavelength (λ_p_). We calculated the plasmon lifetime by using the width of the characteristic resonant peaks at the half-height and the gate voltage at which plasmon resonances arise (see Supporting Information Note 4). The resulting values for τ_p_ were found to be around 0.6 ps, which are larger than the aforementioned scattering time values extracted from the transport analysis. Similarly, the plasmon wavelength can be determined from the measured resonances observed in the photocurrent with respect to the gate voltage and excitation frequency, following the relation^23,28,32^ ω = 2*πs*/λ_p_. The obtained values for λ_p_ range between 600 nm and 2.1 μm (see Figure 2c) for the studied range of THz frequencies (2.5–4.7 THz). These plasmon wavelength values lead to compression ratios (λ_o_/λ_p_, with λ_o_ = *c*/*f* being the wavelength of the incoming THz radiation in free-space) as high as 110 (see details in Supplementary Note 5). The ratio agrees well with the extreme light compression and nanoscale confinement occurring in graphene devices at THz frequencies reported in previous works.^28,32,33^

Finally, we measured the evolution of the plasmonic resonances at 4.7 THz when raising the temperature, *T*. Figure 3a shows the zero-bias photoresponse as a function of the top gate potential for the hole-side (negative *V*_TG_) and the electron-side (positive *V*_TG_) at four selected temperatures within the range 10 K–300 K. Importantly, the observed resonant peaks and dips persist up to 300 K both for electron and hole conduction (see also additional data in Supplementary Note 6). Thus, these measurements demonstrate the resonant detection of THz radiation at room temperature in nonbiased FET devices made from single-layer graphene.

**Figure 3:**
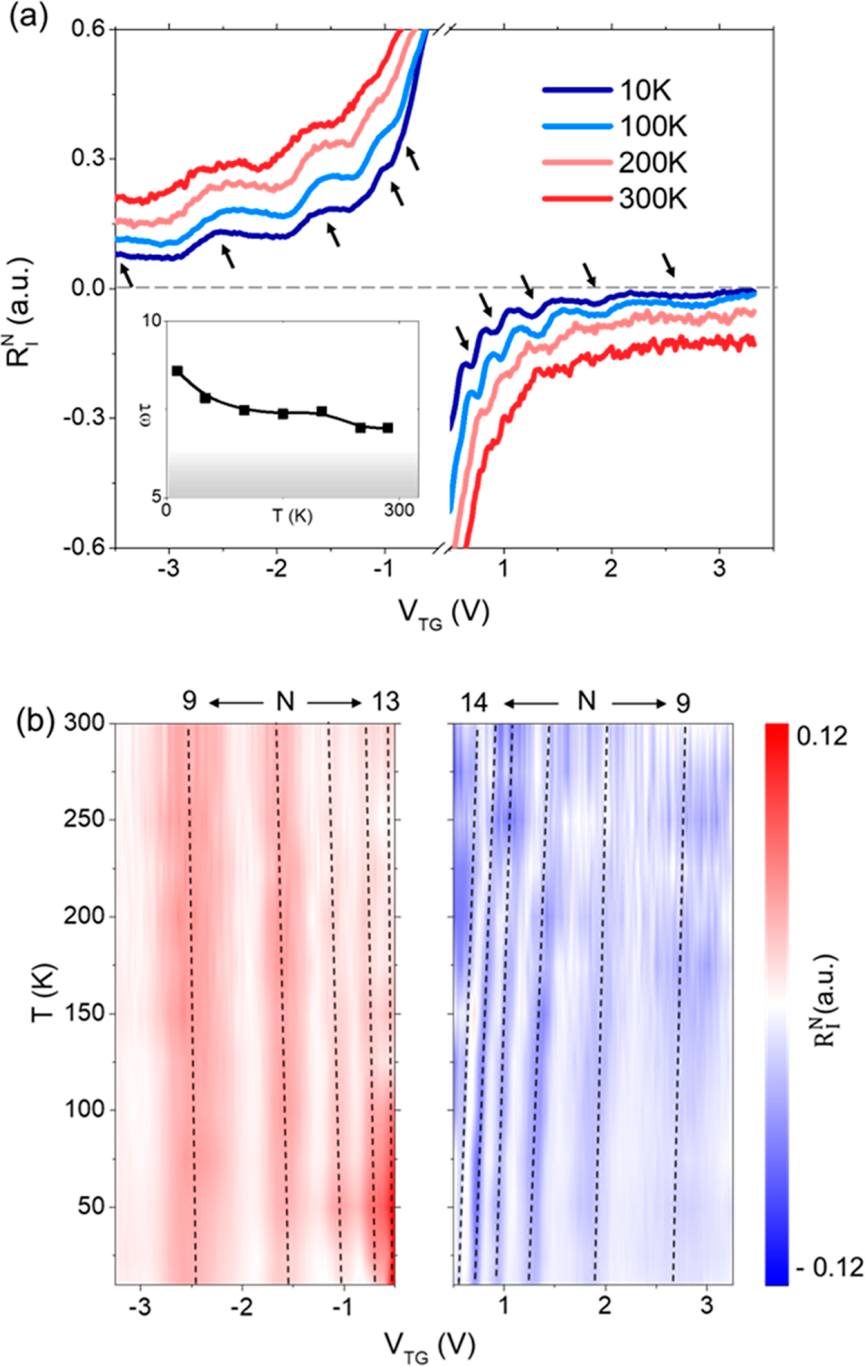
Temperature evolution of the plasmonic resonances. (a) Zero-bias normalized photocurrent as a function of the top gate voltage at four selected temperatures from 10 K up to 300 K (room temperature) at both the hole and electron regions for an incident radiation of 4.7 THz. For an easier visualization, the temperature-dependent photoresponses shown in the panel are normalized with respect to the maximum near the CNP (as done in Figure 2a), and the curves are vertically shifted. The bottom inset shows the temperature evolution of the quality factor *Q = ωτ*. (b) Color mapping of the normalized responsivity, *R*_I_^*N*^ (after subtraction of the nonresonant back-ground), as a function of the top gate voltage at 4.7 THz for all measured temperatures. Vertical dashed lines highlight the evolution of the different observed resonant modes with temperature.

The presence of the photoresponse oscillations with respect to the *V*_TG_ and their detailed evolution with temperature are clearly visible in Figure 3b. In particular, this panel presents the measured photoresponse when excluding (i.e., subtracting) the broadband contribution for all measured temperatures within the range of 10 K–300 K. We stress the fact that resonant peaks and dips appear in the map approximately at the same carrier density (i.e., same value of *V*_TG_) for all temperatures. This observation agrees well with eq 4, which does not contain any explicit dependence of the position of the resonances on *T*. We notice that gate-tunable photoresponse resonances shown in Figure 3a are more evident at the hole side (negative gate voltages) than at the electron side (positive gate voltages). This is also seen in Figure 3b when the resonances are displayed as a function of the temperature. We argue that this could be caused by slightly larger mobilities on the hole side with respect to the electron side in our devices (see Supporting Information Note 2). Moreover, the amplitude of the photocurrent oscillations measured at room temperature in electron or hole conduction depends ultimately on the device (Supporting Information Note 7 showing stronger and more evident resonances measured at room temperature in a second photodetector).

The fact that devices made from high-quality, single-layer graphene exhibit clear and unambiguous evidence of resonant responsivity at room temperature (including the appearance of oscillations of the zero-bias photoresponse with respect to the gate voltage or equivalently carrier density) is extremely relevant for applications. To date, robust signatures of this resonant regime had been reported only to occur at cryogenic temperatures in other semimetals such as bilayer graphene^28^ or 2D electron gases made of III–V semiconductors.^24−26^ Only some experimental indications have been interpreted as arising from resonant detection in III–V field-effect transistors operating at room temperature,^34,35^ but these are vague and rely on the application of a large source-to-drain bias (the application of a source-to-drain dc voltage or current shifts the system toward a resonant regime^36^ but also increases the noise of the rectified signal). In contrast, our study (Figure 3) shows strong and univocal plasmonic resonant oscillations in zero-biased photocurrent measurements performed at room temperature.

We argue that the robust observation of the resonant regime in high-quality single-crystal graphene results from the large room-temperature mobility of the charge carriers in this material,^29,30^ which for our devices is larger than 60000 cm^2^ V^–1^ s^–1^ (see Supplementary Note 2). Such a value leads to a transport scattering time τ = 0.2 ps even at room temperature and to a quality factor *Q* ≫ 1 (*Q* > 6) at an excitation frequency of 4.7 THz (bottom inset of Figure 3a shows the evolution of *Q* with temperature in the device). Since the condition *Q* ≫ 1 is fulfilled, micrometer-size devices made of high-quality monolayer graphene can robustly operate in the weakly damped regime at room temperature and show resonant detection. In contrast, other semiconductor materials have intrinsic carrier mobilities which are around or even below 5000 cm^2^ V^–1^ s^–1^ at room temperature,^37^ which impedes the observation of resonant detection at room temperature. This is even the case of bilayer graphene,^28^ a system which also has lower intrinsic room-temperature mobility values (∼15000 cm^2^ V^–1^ s^–1^) than monolayer graphene due to the presence of additional intrinsic scattering sources including shear phonon scattering^38^ or significantly larger electron–hole collisions.^39^

In summary, we have studied the zero-bias photoresponse of high-mobility monolayer graphene FETs subjected to THz radiation. The operation of the devices is perfectly tuned between nonresonant and resonant regime depending on the frequency of the incoming radiation. In particular, the resonant regime is univocally demonstrated by the measured oscillations present in the gate-voltage-dependent photocurrent. These oscillations are dependent on both the carrier density in the channel and the frequency of the THz radiation. We demonstrate that such univocal fingerprints of resonant THz photodetection are visible not only at cryogenic temperatures but also at room temperature. To the best of our knowledge, this is the first time that resonant THz photodetection has been robustly observed at room temperature without the application of a large drain current bias (which is undesirable for a proper detector operation).

From an application point of view, these findings pave the way for the design and development of a new generation of (graphene-based) plasmonic resonance photodetectors operating at room temperature. The application space of such systems is significant in the THz and mid-infrared regime^6,7^ allowing the realization of emerging and potential technologies at these relatively unexploited but relevant frequencies, including modulators, filters, polarizers, emitters, and selective photodetectors, among many others, as well as the confinement and manipulation of the electromagnetic fields below the classical diffraction limit.^32^

## Supporting Information Available

Additional fabrication details, transport data, calculations, and experimental results (PDF)
Two supporting movies that show plasma-waves propagating in plasmonic cavities (MP4-1, MP4-2)
