# Supplementary material for: Room-Temperature Plasmon-Assisted Resonant THz Detection in Single-Layer Graphene Transistors
Source: Nano Lett. 2024 Jan 2;24(3):935–42. doi: 10.1021/acs.nanolett.3c04300 (PMC10811671; doi:10.1021/acs.nanolett.3c04300)
Supplement: Supplementary file 3 — nl3c04300_si_003.pdf [file nl3c04300_si_003.pdf]

Supplementary Information for "Room-Temperature Plasmon-Assisted Resonant THz Detection in Single-Layer Graphene Transistors"

José M. Caridad[1,2], Óscar Castelló[1,2], Sofía M. López Baptista[1], Takashi Taniguchi[3], Kenji Watanabe[4], Hartmut G. Roskos[5], Juan A. Delgado-Notario[1*]

[1] Department of Applied Physics, University of Salamanca, Salamanca, 37008, Spain

[2] Unidad de Excelencia en Luz y Materia Estructurada (LUMES), Universidad de Salamanca, Salamanca, 37008, Spain

[3] Research Center for Materials Nanoarchitectonics, National Institute for Materials Science, 1-1 Namiki, Tsukuba, 305-0044, Japan

[4] Research Center for Electronic and Optical Materials, National Institute for Materials Science, 1-1 Namiki, Tsukuba, 305-0044, Japan

[5] Physikalisches Institut, Johann Wolfgang Goethe-Universität, Max-von-Laue-Str. 1, Frankfurt am Main, D-60438, Germany

*corresponding author: juanandn@usal.es

Supplementary Note 1. Fabrication details

Monolayer graphene and hexagonal-boron-nitride (hBN) were mechanically exfoliated on a Si substrate with 300 nm of SiO_2 thermally growth and identified via optical contrast using an optical microscope. The exfoliated hBN flakes were also characterized by a Stylus Profilometer (Bruker DektakXT®) to estimate their thickness, obtaining values of 28 nm for the top and 32 for the bottom. The graphene-based heterostructure was fabricated by using a state-of-the-art dry-stacking technique[1,2] to encapsulate the monolayer graphene in between two thin hBN flakes (See Figure S1). Importantly, we have intentionally selected a large and straight graphene flake to naturally define the width, W, of device channel ($W \approx 4.5$ µm). Initially, the sample was patterned via electron beam lithography (EBL) and dry etching in a SF_6 atmosphere (20 ºC, 40 sccm and 75W) to remove unwanted flakes transferred near the graphene-based stack. Then, Drain and Source side metallic contacts and the bowtie antenna were fabricated via EBL, dry etching in a SF_6 atmosphere (10 ºC, 40 sccm and 75W) and e-beam evaporation of 3.5 nm Cr and 55 nm Au. Here one of the bows of the antenna was connected to the Source contact. Finally, another round of EBL and e-beam evaporation of 5 nm Cr and 40 nm Au was undertaken to fabricate the Top Gate and connect it to the other bow of the antenna.

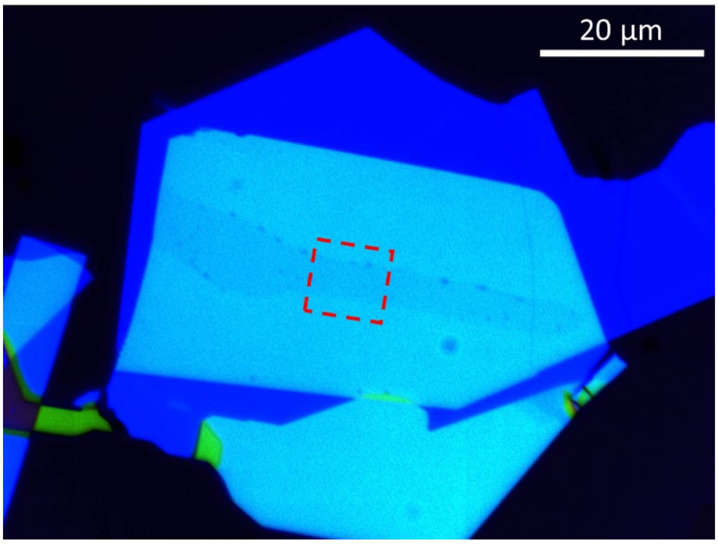

Figure S1. Optical image of the hBN/Monolayer Graphene/hBN stack**.** Red-dashed line highlights the area selected for the fabrication of the Terahertz detector.

Supplementary Note 2. Transport measurements

Two-terminal dc transport measurements were performed via lock-in technique where a pseudo-dc current (10 nA at 11.33 Hz) was injected in the device and the generated voltage drop across source and drain electrodes was recorded by a lock-in amplifier (SR860). The applied top gate potential was generated with a dc voltage generator (Keithley 2614B). Figure S2 (a) shows the two-terminal channel resistance, r_{ch}, w.r.t the top gate potential, V_{TG}, measured at several, equally spaced temperatures in the range 10K-300K. Instead, Figure S2 (b) shows the channel resistance curves at four selected temperatures. The measured resistance exhibits a bell shape characteristic of graphene devices[2–5], with the resistance maxima occurring at the charge neutrality point (CNP) and a decrease in magnitude when applying a top gate potential away of the CNP.

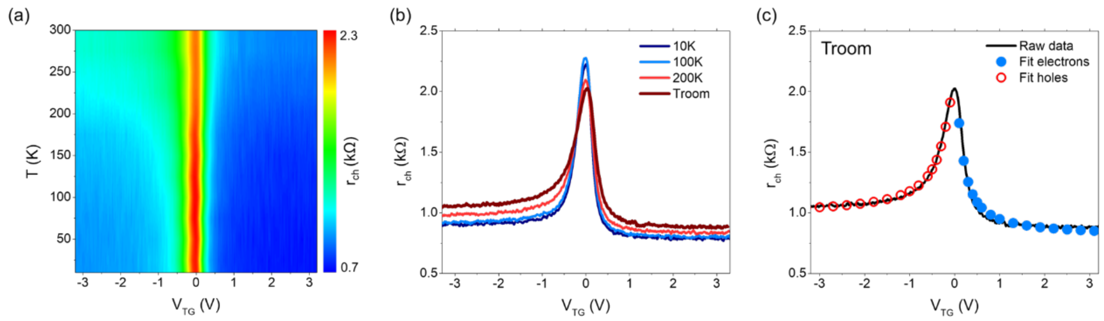

Figure S2. (a) Two-terminal resistance, r_{ch}, w.r.t. the top gate voltage measured at several temperatures. (b) Two-terminal resistance w.r.t. the top gate potential at four selected temperatures extracted from panel (a). (c) Experimental (black line) and fitted (blue and red dots) channel resistance data at room temperature

In order to estimate average values of carrier mobility in our device, we have fitted the two-terminal resistance curves following the model presented by L. Gammelgaard and co-authors[6] characterized by the equation:

$$r_{ch} = R_c + \frac{L/_W}{\sqrt{\left(\frac{L/_W}{(R_{CPN} - R_c)e\mu}\right)^2 + n^2\, e\mu}} \tag{1}$$

where R_c is the parasitic resistance (i.e. the two contact resistances and the resistance of the ungated regions in the graphene channel), L and W are the channel length and width respectively, R_{CNP} is the resistance value at the CNP, e is the elementary charge, μ is the carrier mobility and n is the carrier concentration approximated by $n = C_{ox}V_{TG}^{*}/e$ where C_{ox} is the gate oxide capacitance per unit area and V_{TG}^{*} is the applied top gate voltage with respect to the CNP. We have obtained values of average carrier mobilities exceeding 70000 $cm^2V^{-1}s^{-1}$ at temperatures around 10 K and remain as high as 60000 $cm^2V^{-1}s^{-1}$ at room temperature.

To underpin the high-mobility values of charge carriers in our samples, we have additionally fabricated a multi-terminal Hall bar device using the same procedure as described in the manuscript and Supplementary Note 1. The longitudinal device resistance was measured via four-terminal configuration at different back-gate voltages (see Figure S3). The field-effect mobility μ was calculated using the Drude model of conductivity given by the formula $\sigma = \mu_i e n_i$, where σ is the is the electrical conductivity, e is the elementary charge and n_i is the electron or hole carrier densities. We observed maximum carrier mobilities exceeding 150000 cm^2/Vs for holes and electrons in the samples at 10K (see Figure S3).

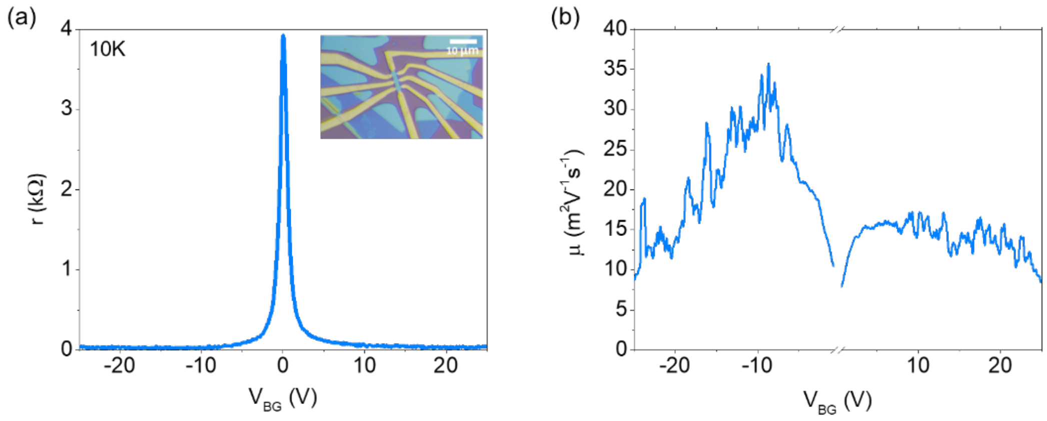

Figure S3. (a) 4-terminal resistance, r, as a function of the back-gate voltage, V_{BG}. Inset panel shows the optical photograph of the Hall bar device. (b) Carrier mobility as a function of the back-gate potential. Temperature was fixed at 10K.

Supplementary Note 3. Additional frequency dependence data

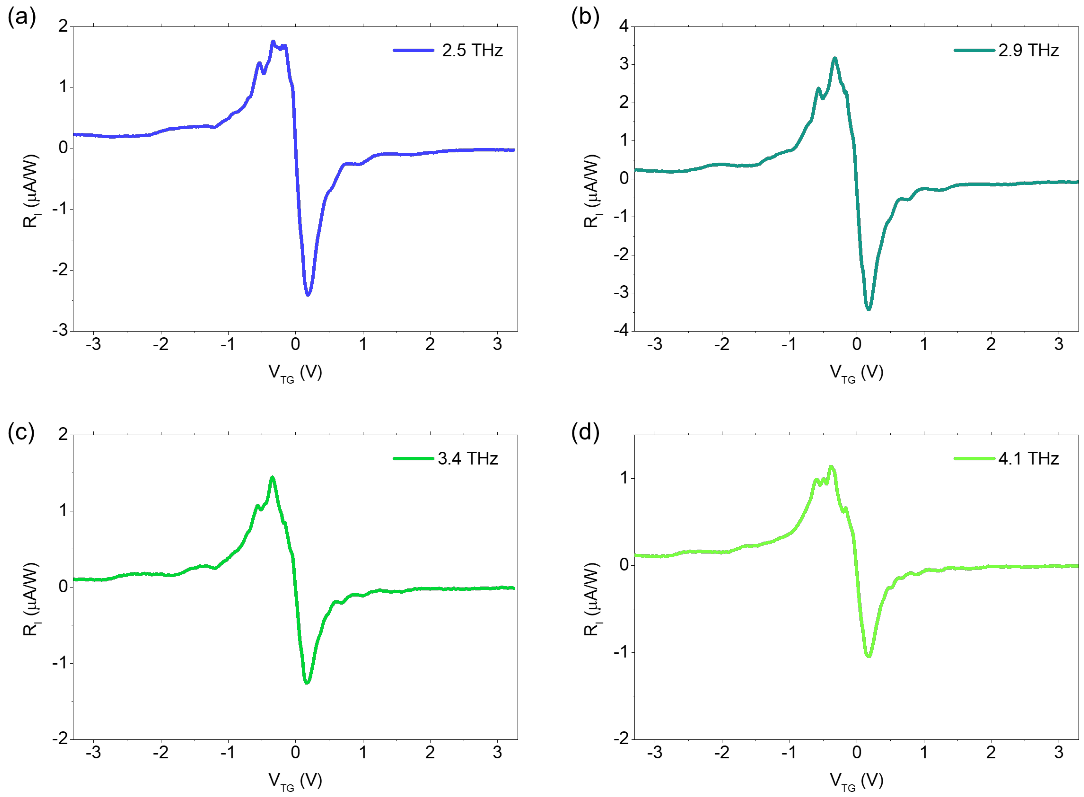

Figure S4. Current responsivity, R_I, as a function of the top gate voltage, V_{TG}, measured for an incoming radiation of (a) 2.5 THz, (b) 2.9 THz, (c) 3.4 THz and (d) 4.1 THz. Temperature was fixed at 10K in all the measurements.

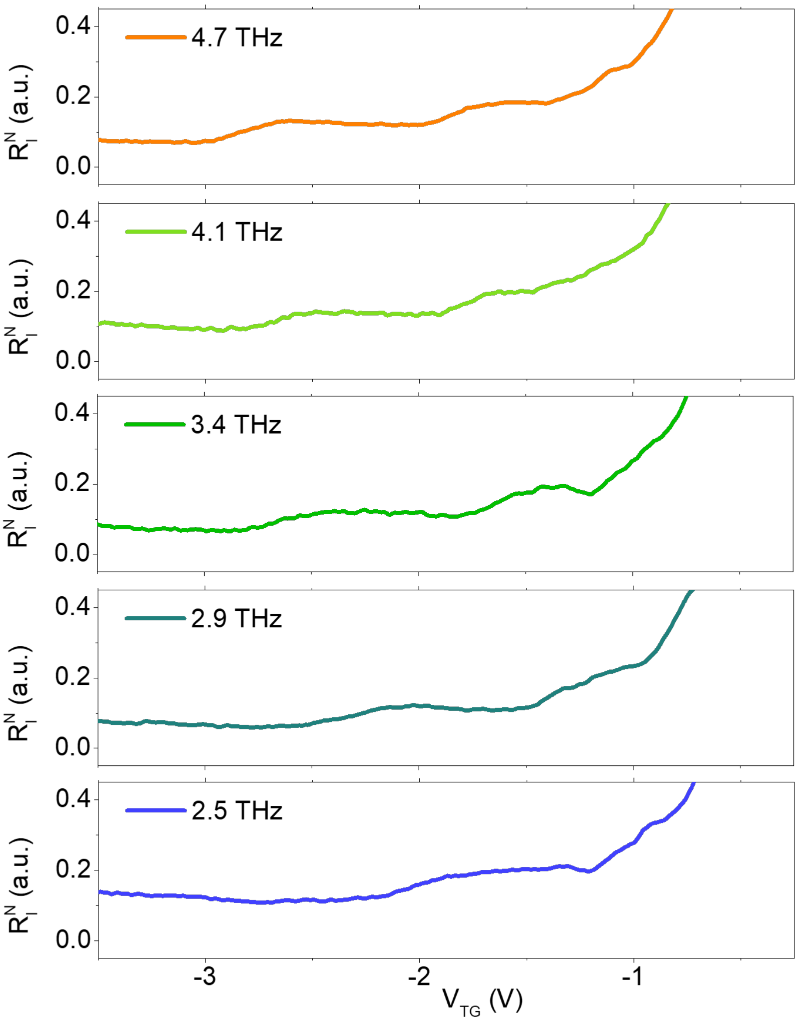

Figure S5. Zoomed normalized current responsivity, R_I^N, as a function of the top gate voltage, V_{TG}, at the hole side for a set of incoming frequencies in the resonant regime (2.5 THz - 4.7 THz, all frequencies making $Q \gg 1$). All measurements in these five panels were performed at 10K.

Supplementary Note 4. Graphene plasmons in single-layer graphene FET devices

As stated in the main text, the resonant mode number in monolayer graphene with the applied gate voltage follows the formula:

$$N = \frac{L_G \omega}{\pi \sqrt{\frac{e\, v_F}{\hbar}\left(\frac{e}{\pi C_{OX}}\right)^{\frac{1}{4}}}} |V_G|^{-\frac{1}{4}} - \frac{1}{2} \tag{2}$$

Which predict a linear dependence of the different resonant modes accessible in our plasmonic cavity with respect to $|V_G|^{-1/4}$, the frequency ω and the cavity length, L_G.

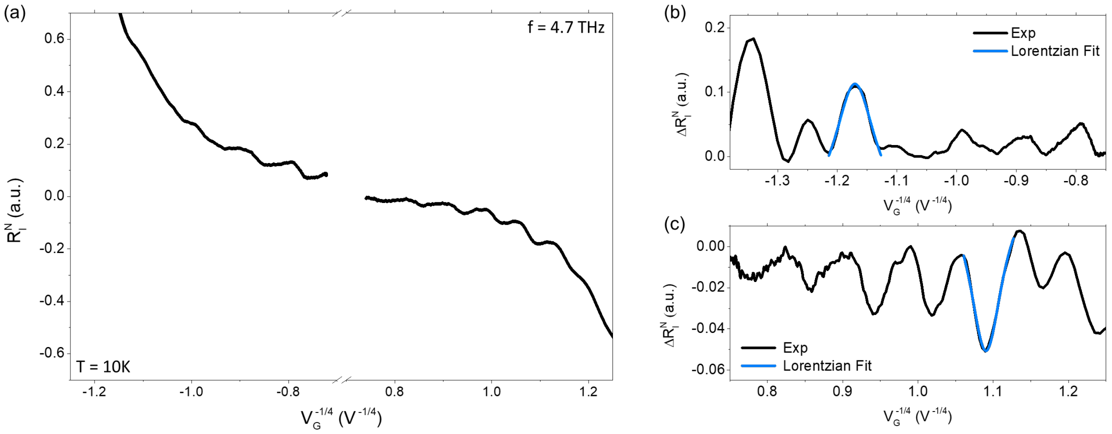

Figure S6. (a) Normalized current responsivity, R_I^N, as a function of $V_G^{-1/4}$ for an excitation frequency of 4.7 THz at 10K. (b) and (c) Experimental normalized current responsivity (black line) upon removing the non-resonant background as a function of $V_G^{-1/4}$ for the hole side (b) and electron side (c). Solid blue lines correspond to the Lorentzian fitting with a FWHM = 0.056 $V^{-1/4}$ for the electron side and FWHM = 0.085 $V^{-1/4}$ for the hole side which corresponds to τ = 0.6 ps and τ = 0.47 ps respectively for an incoming 4.7 THz.

First, we have analyzed the tunability of the different resonant modes with $|V_G|^{-1/4}$ for an incoming THz radiation of 4.7 THz at 10K. Figure S5 (a) shows the normalized current responsivity as a function of $|V_G|^{-1/4}$. Figure S5(b) and (c) show a similar graph where, for clarity, the non-resonant signal back-ground in the current responsivity has been removed. As expected, the emerged oscillations (corresponding to the different resonant modes appearing at the hole and electron sides) are located equidistantly with respect to the increase of $|V_G|^{-1/4}$ in clear agreement with equation (2). Moreover, we obtain the plasmon lifetime, τ_p from panels (b) and (c). Adapting the theory developed by Bandurin and coworkers for the case of bilayer graphene[7], $\tau_p$in the case of single-crystal graphene can be extracted from the peak-width at the half-heigh according to:

$$\frac{FWHM}{V_G^{-1/4}} = \frac{1}{\omega\tau} \tag{3}$$

By using a Lorentzian fit in the current responsivity curves (see Figure S5 (b)) τ_p were found to be around 0.6 ps for the electron side and 0.47 ps for the hole side. These values are of the same order of magnitude than the momentum relaxation time extracted from transport analysis but slightly larger ($\tau \approx 0.29$ ps).

Moreover, we have performed simulations analysis of the dependence of the plasmonic cavity length to observe the propagation of plasma waves in time (See supporting movies 1 and 2). When the plasmonic cavity length is much larger than the plasmon propagation length (i.e. $L_G >> L_P$), propagating plasmons in graphene decay before reach the end of the cavity (See supplementary movie 2). In this scenario, the rectified photocurrent do not differ for both resonant (Q >> 1) or non-resonant regime (Q << 1, case of our device at the measured frequency 0.3THz), and hence no frequency and gate dependent oscillations appear in the photoresponse. When $L_G \approx L_P$, (case of our device at frequencies > 2.5THz)

propagating plasma waves slightly decay and they can reach the end of the channel, be reflected and therefore creating a constructive and destructive interferences in the channel in a quasi-standing wave scenario. In this regime, a notable dependence of the photoresponse on the incoming THz frequency and the applied gate voltage is observed (See supplementary movie 2).

Supplementary Note 5. THz field confinement in FET devices made of single-layer Graphene

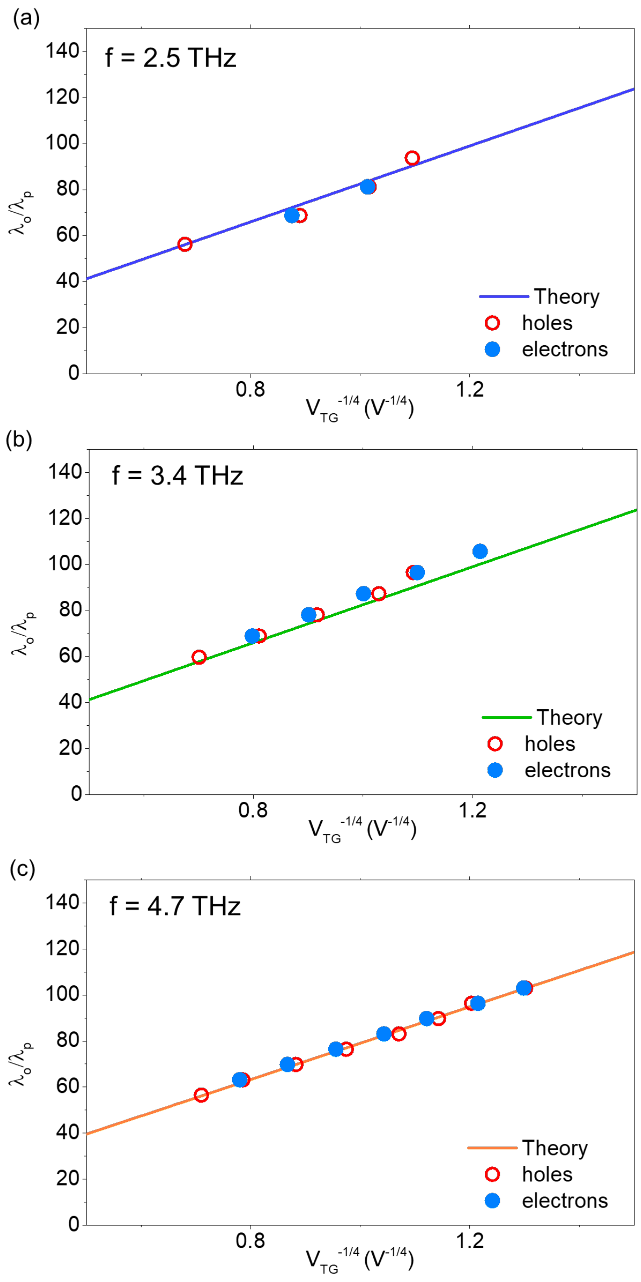

Figure S7. Compression ratio, λ_o/λ_p, as a function of $V_{TG}^{-1/4}$ for excitation frequencies of 2.5 THz (a), 3.4 THz (b) and 4.7 THz (c) at 10K.

Supplementary Note 6. Room temperature resonant THz photodetection

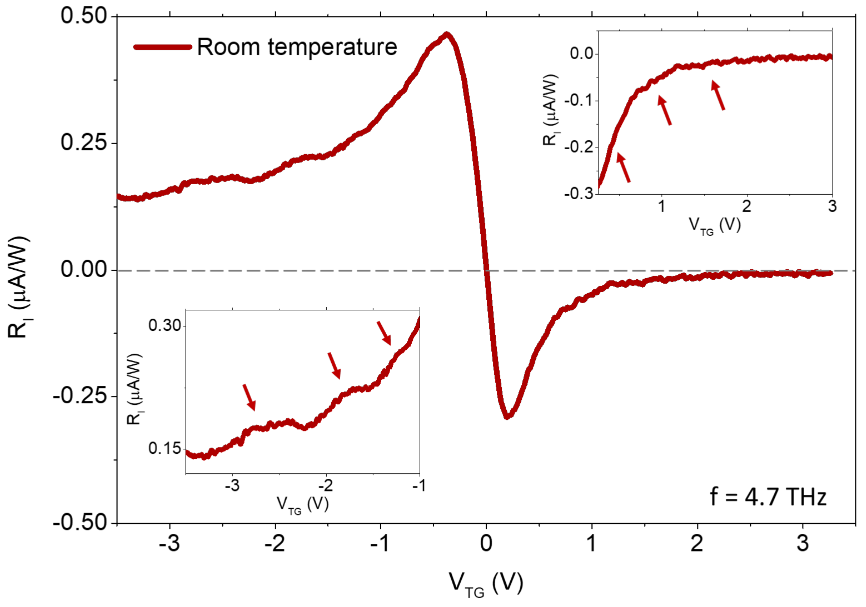

Figure S8. Current responsivity, R_I, as a function of the top gate voltage, V_{TG}, measured at 4.7 THz at room temperature. Inset panels show a zoomed area of the recorded current responsivity for electron (upper-right) and hole (bottom-left) carriers where resonant peaks are highlighted by red arrows.

Supplementary Note 7. Additional THz photodetectors

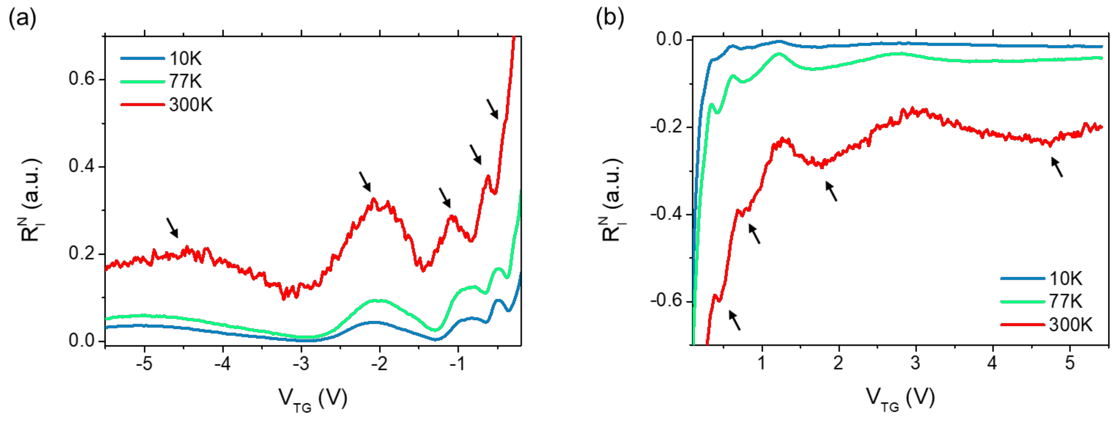

Figure S9. Additional device showing room-temperature resonant photoresponse. Zero-bias normalized photocurrent as a function of the top gate voltage for three selected temperatures of 10 K, 77K and 300K (room temperature) at the hole (a) and electron (b) regions for an incident radiation of 2.5 THz for a second device with L = 4 μm. For an easier visualization, the temperature dependence photoresponses shown in the panel are normalized with respect to the maxima near the CNP.

References

(1) Purdie, D. G.; Pugno, N. M.; Taniguchi, T.; Watanabe, K.; Ferrari, A. C.; Lombardo, A. Cleaning Interfaces in Layered Materials Heterostructures. *Nat Commun* **2018**, *9* (1), 5387. https://doi.org/10.1038/s41467-018-07558-3.

(2) Delgado-Notario, J. A.; Knap, W.; Clericò, V.; Salvador-Sánchez, J.; Calvo-Gallego, J.; Taniguchi, T.; Watanabe, K.; Otsuji, T.; Popov, V. V; Fateev, D. V; Diez, E.; Velázquez-Pérez, J. E.; Meziani, Y. M. Enhanced Terahertz Detection of Multigate Graphene Nanostructures. **2022**, *11* (3), 519–529. https://doi.org/doi:10.1515/nanoph-2021-0573.

(3) Kim, S.; Nah, J.; Jo, I.; Shahrjerdi, D.; Colombo, L.; Yao, Z.; Tutuc, E.; Banerjee, S. K. Realization of a High Mobility Dual-Gated Graphene Field-Effect Transistor with Al2O3 Dielectric. *Appl Phys Lett* **2009**, *94* (6), 062107. https://doi.org/10.1063/1.3077021.

(4) Bandurin, D. A.; Gayduchenko, I.; Cao, Y.; Moskotin, M.; Principi, A.; Grigorieva, I. V; Goltsman, G.; Fedorov, G.; Svintsov, D. Dual Origin of Room Temperature Sub-Terahertz Photoresponse in Graphene Field Effect Transistors. *Appl Phys Lett* **2018**, *112* (14), 141101. https://doi.org/10.1063/1.5018151.

(5) Vaquero, D.; Clericò, V.; Schmitz, M.; Delgado-Notario, J. A.; Martín-Ramos, A.; Salvador-Sánchez, J.; Müller, C. S. A.; Rubi, K.; Watanabe, K.; Taniguchi, T.; Beschoten, B.; Stampfer, C.; Diez, E.; Katsnelson, M. I.; Zeitler, U.; Wiedmann, S.; Pezzini, S. Phonon-Mediated Room-Temperature Quantum Hall Transport in Graphene. *Nat Commun* **2023**, *14* (1), 318. https://doi.org/10.1038/s41467-023-35986-3.

(6) Gammelgaard, L.; Caridad, J. M.; Cagliani, A.; MacKenzie, D. M. A.; Petersen, D. H.; Booth, T. J.; Bøggild, P. Graphene Transport Properties upon Exposure to PMMA Processing and Heat Treatments. *2d Mater* **2014**, *1* (3), 035005. https://doi.org/10.1088/2053-1583/1/3/035005.

(7) Bandurin, D. A.; Svintsov, D.; Gayduchenko, I.; Xu, S. G.; Principi, A.; Moskotin, M.; Tretyakov, I.; Yagodkin, D.; Zhukov, S.; Taniguchi, T.; Watanabe, K.; Grigorieva, I. V; Polini, M.; Goltsman, G. N.; Geim, A. K.; Fedorov, G. Resonant Terahertz Detection Using Graphene Plasmons. *Nat Commun* **2018**, *9* (1), 5392. https://doi.org/10.1038/s41467-018-07848-w.
